# Self-reported oral health problems and the ability to organize dental care of community-dwelling elderly aged ≥75 years

**DOI:** 10.1186/s12903-020-01175-7

**Published:** 2020-07-02

**Authors:** M. H. Bakker, A. Vissink, S. L. W. Spoorenberg, K. Wynia, A. Visser

**Affiliations:** 1 Department of Maxillofacial Surgery, University of Groningen, University Medical Center Groningen, PO Box 30.001, NL-9700 RB Groningen, The Netherlands; 2 Department of Health Sciences, Community and Occupational Medicine, University Medical Center Groningen, University of Groningen, Groningen, the Netherlands; 3 Department of Geriatric Dentistry, Dental School, Center for Dentistry and Oral Hygiene, University of Groningen, University Medical Center Groningen, Groningen, the Netherlands

**Keywords:** Ageing, Oral health problems, Dental care use, Oral pain, Community-dwelling elderly

## Abstract

**Background:**

It is unclear how many community-dwelling elderly (≥75 years) experience oral health problems (e.g. pain, dry mouth, chewing problems) and how they manage their dental care needs. This study aimed to assess self-reported oral health problems in elderly who are frail or have complex care needs, and their ability to organize dental care when reporting oral pain.

**Methods:**

Three thousand five hundred thirty-three community-dwelling elderly participating in the “Embrace” project were asked to complete questionnaires regarding oral status and oral health problems. Frailty was assessed with the Groningen Frailty Indicator (GFI). Intermed for Elderly Self-Assessment (IM-E-SA) was used to determine complexity of care needs. Next, elderly who reported oral pain were interviewed about their oral pain complaints, their need for dental care, and their ability to organize and receive dental care. For statistical analyses Chi^2^-tests and the one-way ANOVA were used.

**Results:**

One thousand six hundred twenty-two elderly (45.9%) completed the questionnaires. Dry mouth (11.7%) and oral pain (6.2%) were most frequently reported. Among the elderly reporting oral pain, most were registered at a local dentist and could go there when needed (84.3%). Robust elderly visited the dentist independently (87%), frail (55.6%) and complex (26.9%) elderly more often required assistance from caregivers.

**Conclusions:**

Dry mouth and oral pain are most reported oral health problems among community-dwelling elderly. Elderly with complex care needs report most oral health problems. In case an elderly seeks dental treatment to alleviate an oral pain complaint, most elderly in this study were able to organize dental care and transport to the dentist. Frail and complex elderly often need assistance from caregivers to visit the dentist. Therefore caretakers should keep in mind that when frailty progresses, visiting a dentist may become more and more difficult and the risk for poor oral health increases.

## Background

Globally the population is growing and aging [1, 2]. This development will have great impact on all healthcare systems. As people grow older, staying vital and healthy becomes challenging as elderly increasingly become frail and care-dependent [3]. Frailty is defined as a state in which older adults are vulnerable to sudden changes in health status because of a decline in physiological function and reserve [3]. Recent studies have shown that the prevalence of multimorbidity and polypharmacy rapidly increases with age, resulting in complex care needs in elderly [4, 5]. Complex care needs may arise when elderly are suffering from multiple chronic diseases and polypharmacy and are treated by various medical healthcare professionals [6].

In the last two decades, as a result of improved dental care in the previous century, edentulism is decreasing and more and more elderly retain their own teeth until high age or receive dental implants [7]. Elderly with remaining teeth and elderly provided with dental implants to support their overdentures have a high risk of oral problems, especially when oral hygiene maintenance and dental visits become difficult due to frailty [8, 9]. But even full dentures can become problematic when the denture fit is poor [8]. Frail and care-dependent elderly therefore have a relatively high risk of poor oral health and subsequently of oral pain (pain originating from oral tissues) [10]. This is a great hazard as poor oral health and oral pain have a negative effect on general health and quality of life, and can limit social interactions [11, 12].

Regular dental visits are therefore advised in order to prevent poor oral health. Research in the United States (US) has shown that only 46% of community-dwelling elderly visit the dentist for a general check-up, and this figure decreases as these elderly get older and subsequently become more frail [13]. When elderly can no longer live independently at home, they are often admitted in a nursing home. Studies showed that after dental examination 70% of the residents were in need of dental treatment [14, 15]. Oral pain among community-dwelling elderly are described in literature [16–18]. For example, Hoeksema et al. [19] reported high a prevalence (22%) of oral pain among community-dwelling elderly. However, the proportion of community-dwelling elderly who experience oral health problems such as dry mouth, oral pain and chewing problems and if these elderly are able to manage their dental care – especially when suffering from oral pain – remains unclear. Therefore, the aim of this study was to assess self-reported oral health problems (such as oral dryness, pain, chewing problems) in community-dwelling elderly (aged ≥75 years) who are frail or have complex care needs. Next, it was assessed if and how these elderly are able to organize dental care when suffering from oral pain.

## Methods

### Participants and study design

We asked all community-dwelling elderly (aged ≥75 years) living in the northern region of the Netherlands who were participating in the ongoing Embrace program for person-centered care to participate in our study [20–23]. The Embrace program (“SamenOud” [aging together] in Dutch) focuses on elderly patients of general practitioners (GPs). Embrace is an integrated care service aimed to prolong the ability of older adults to age at home for as long as possible by providing comprehensive, coherent, person-centered, proactive, and preventive care and support. For an extensive description of the program see Spoorenberg et al. and Uittenbroek et al. [20–23].

The Medical Ethical Committee of the University Medical Center Groningen (the Netherlands) approved the Embrace study proposal (reference METc2011.108). Regarding the present study, they concluded that additional approval for assessing perceived oral health and the need for treatment was not required. The study was performed in accordance with the principles expressed in the Declaration of Helsinki.

### Procedures and assessments

Between July 2017 and February 2018, a total of 3533 community-dwelling elderly participating in Embrace and living in the northern parts of the Netherlands received self-reporting questionnaires regarding demographics (age, sex, general health (underlying diseases, use of drugs)). In addition, oral status (remaining teeth (including fixed implant-retained structures), edentulous with a conventional denture or removable implant-retained overdentures (IOD)), and oral health problems (oral pain, chewing problems, swallowing problems, dry mouth, feeling of insecurity regarding their oral status) experienced in the last 3 months were scored.

The elderly also completed a number of validated health-related questionnaires:

- *Groningen Frailty Indicator* (GFI): assesses physical and psychological frailty among elderly. This valid and reliable 15-item instrument results in a score ranging from 0 to 15, with higher scores corresponding with a higher level of frailty. A score of ≥4 is regarded as frail [24].
- The *INTERMED for the Elderly Self-Assessment* (IM-E-SA): assesses the need for complex care of elderly. This valid and reliable instrument [6] consists of 20 questions in four domains (biological, psychological, social and healthcare), and it provides insight in perceived physical and cognitive abilities as well as healthcare needs. Scoring ranges from 0 to 60, with a higher score corresponding to a higher need for complex care. A cut-off value of ≥16 was used to define elderly in need for complex care [6].

Elderly who completed all questionnaires were included in this study. Elderly with incomplete questionnaires were excluded from this study.

### Case complexity

Participating elderly were categorized based on their IM-E-SA and GFI scores in three groups; 1) robust elderly, 2) frail elderly and 3) elderly with complex care needs. Robust elderly were defined as resilient persons in good health. Robust elderly showed low levels of frailty (GFI < 4) and a low level of complex care needs (IM-E-SA < 16). Frail elderly were defined as having a higher level of frailty (GFI ≥ 4), but a low level of complex care needs (IM-E-SA < 16). Elderly with complex care needs were characterized by a high IM-E-SA score (IM-E-SA ≥16).

### Interview on oral pain

Elderly reporting oral pain were included for further research. Studies have shown that oral pain is a strong motivator to visit the dentist [25, 26], but it is unclear whether community-dwelling elderly are able to visit the dentist when they are suffering from oral pain. In this study researcher (MHB) contacted elderly with oral pain in the last 3 months by telephone for a structured interview. This was done within 2 weeks after the questionnaire had been returned. This interview was held to obtain additional information on the reported pain complaints and how these elderly organize their dental care needs. Information acquired by the interview:

- Actual status of reported oral pain;
- Severity of actual pain on a visual analog scale of 1 to 10, where 10 indicates severe pain;
- Etiology of oral pain;
- History of oral pain;
- Location of oral pain;
- Medication and actions the participant had already taken regarding the reported oral pain (e.g., taking painkillers, visiting a dentist, using mouthwash, additional dental cleaning, consulting friends, visiting a general practitioner);
- Regular visits to the dentist;
- Transport to the dentist.

In case oral pain was still present and the dentist or a specialist had not been visited thus far, the research team advised the patient to visit a dentist. After the structured interview all participants received a letter containing a short summary of the interview including the given advice for organizing an appointment with the dentist.

If the oral pain complaint was complicated (e.g., burning mouth syndrome, pain related to previous head and neck oncology treatment) and the earlier consulted dentist could not alleviate the pain complaint, the patient was advised to return to the dentist and inform whether it would be possible to be referred to a maxillofacial surgeon. Elderly who were advised to visit a dentist or specialist were contacted again after 6–8 weeks. In this second interview, the participants were asked whether they had visited a dentist or maxillofacial surgeon and what the current status of their oral pain complaint was. Elderly were excluded when they did not give consent to the interview or when they could not be contacted or did not answer the telephone.

### Statistical analysis

Data analysis was performed with IBM SPSS Statistics 23 (SPSS Inc., IBM Company, IBM Corporation, Chicago, IL, US). A significance level of *p* < 0.05 was chosen for all tests. The Shapiro-Wilkins test was used to assess normality of the data (p < 0.05). Median and interquartile ranges were provided for the not normally distributed clinical parameters. Mean and standard deviation were used for normally distributed parameters. Chi^2^ tests were used to assess significant differences between elderly with different risk profiles. For normally distributed variables one-way ANOVA was used, post hoc analysis was performed using independent-samples t-test. *P* < 0.05 was determined as cut-off value. Because interviews were used to assess oral pain, no missing data were encountered.

## Results

### Respondents

**Fig. 1:**
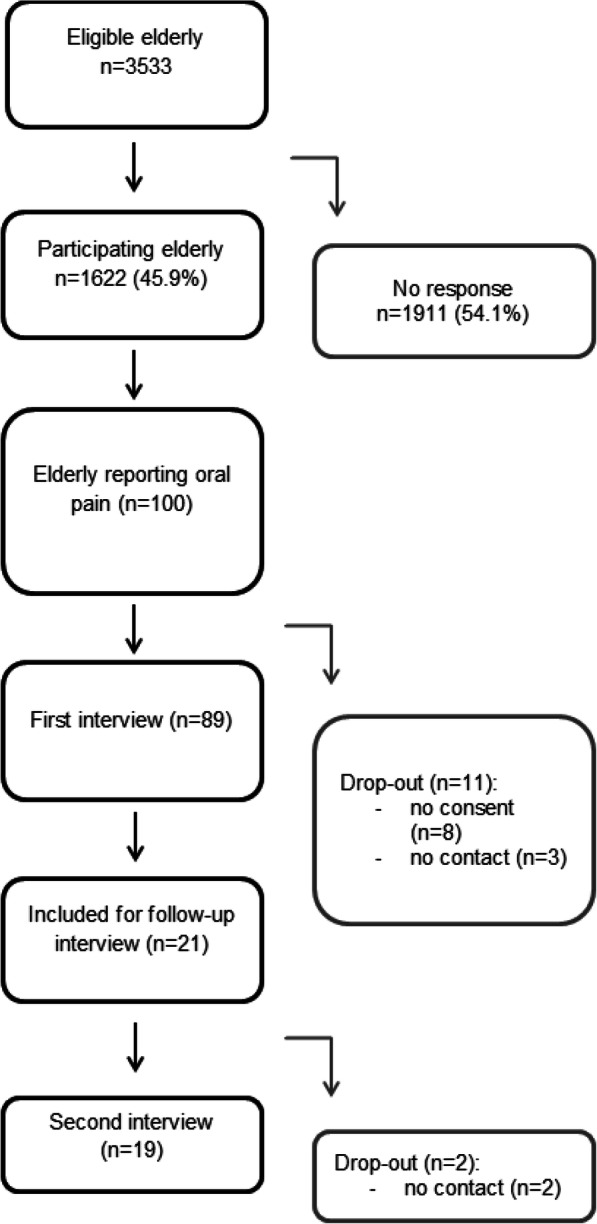
Flow diagram of patient inclusion process

Demographics of the respondents are shown in Table 1. Next, the flowchart of this study is shown in Fig. 1. All 3533 elderly who participated in Embrace were eligible and invited to join this study. In total, 1622 elderly (45.9%) returned the questionnaires. Dry mouth (11.7%) and oral pain (6.2%) were most reported oral health problems. Elderly with complex care needs reported most frequently oral pain, dry mouth, swallowing problems, chewing problems and an insecure feeling. Elderly who reported oral pain (*n* = 100, 6.2%) were telephoned within 2 weeks after the questionnaire had been returned and invited to participate in an additional structured interview, of which 89 (89%) responded positively (8 elderly decided not to participate in the interview, and 3 elderly could not be contacted).

**Table 1:** Patient characteristics

*Patient characteristics (n,%)*	*Total* *(n = 1622)*	*Robust* *(n = 1133)*	*Frail* *(n = 226)*	*Complex* *(n = 263)*	*P-value between groups*
Female	897 (55.3%)	597 (52.7%)^a^	145 (64.2%)	155 (58.9%)	0.003
Age (mean, SD)	82 (4.4)	82 (4.5)^a,b^	83 (3.9)	82 (4.1)	0.023
*Oral status*					
Edentulous	722 (44.5%)	479 (42.3%)^b^	100 (44.2%)	143 (54.4%)	0.002
Implant overdenture	189 (11.7%)	131 (11.6%)	27 (11.9%)	31 (11.8%)	0.984
Remaining teeth	711 (43.8%)	523 (46.2%)^b^	99 (43.8%)	89 (33.8%)	0.001
*Oral health problems*					
Oral pain	100 (6.2%)	58 (5.1%)^b^	15 (6.6%)	27 (10.3%)	0.007
Chewing problems	92 (5.7%)	52 (4.6%)^b^	14 (6.2%)	26 (9.9%)	0.003
Swallowing problems	36 (2.2%)	14 (1.2%)^b^	6 (2.7%)	16 (6.1%)	< 0.001
Dry mouth	190 (11.7%)	105 (9.3%)^a,b^	34 (15%)	51 (19.4%)	< 0.001
Insecurity	77 (4.7%)	41 (3.6%)^c^	12 (5.3%)	24 (9.1%)	0.001
*Total number of oral health problems*					
1 problem	288 (17.8%)	163 (14.4%)^a,b^	56 (24.8%)	69 (26.2%)	< 0.001
2 problems	73 (4.5%)	38 (3.4%)^b^	11 (4.9%)	24 (9.1%)	< 0.001
3 problems	17 (1.0%)	7 (0.6%)^b^	1 (0.4%)	9 (3.4%)	< 0.001
4 problems	–	–	–	–	–
5 problems	2 (0.1%)	2 (0.2%)	–	–	0.386

^a^ Statistically significant difference (p < 0.05) between robust and frail elderly

^b^ Statistically significant difference (*p* < 0.05) between robust and complex elderly

### First interview

Of the 100 respondents reporting oral pain, 32.6% (*n* = 29) were still suffering from oral pain at the time of the interview, and 38.2% (*n* = 34) reported that the pain had or has lasted for over 6 weeks (Table 2). There were no statistically significant differences between respondents with different risk profiles. In order to relieve their oral pain, 56.2% of the elderly had already visited the dentist.

**Table 2:** First semi-structured interview

*First interview (n,%)*	*Total* *(n = 89)*	*Robust* *(n = 54)*	*Frail* *(n = 9)*	*Complex* *(n = 26)*	*P-value between groups*
Experiencing oral pain at time of interview	29 (32.6%)	16 (29.6%)	2 (22.2%)	11 (42.3%)	0.412
Oral pain lasted > 6 weeks	34 (38.2%)	19 (35.2%)	3 (33.3%)	12 (46.2%)	0.631
VAS^1^ pain score (mean, SD)	5.8 (2.1)	6.1 (2.5)	6.0 (1.4)	5.5 (1.9)	0.855
No pain complaint or cannot remember	9 (10.1%)	5 (9.3%)	1 (11.1%)	3 (11.5%)	0.881
*Measures taken to relieve oral pain complaints*					
Visited the dentist for oral pain complaint	50 (56.2%)	35 (64.8%)	3 (33.3%)	12 (46.2%)	0.100
Other measures (painkillers, mouth rinse)	9 (10.1%)	6 (11.1%)	1 (11.1%)	2 (7.7%)	1.000
*Type of oral pain complaint*					
Toothache	21 (23.6%)	15 (27.8%)	2 (22.2%)	4 (15.4%)	0.471
Denture-related complaint	22 (24.7%)	10 (18.5%)	3 (33.3%)	9 (34.6%)	0.241
Minor dental complaint	21 (23.6%)	12 (22.2%)	2 (22.2%)	7 (26.9%)	0.893
Complicated pain complaints	6 (6.7%)	6 (11.1%)	–	1 (3.8%)	0.212
Periodontal disease (increased tooth mobility)	4 (4.5%)	3 (5.6%)	1 (11.1%)	1 (3.8%)	1.000
Peri-implant pain (peri-implant tissue)	4 (4.5%)	2 (3.7%)	–	1 (3.8%)	0.545
Fractured teeth, radix relicta	2 (2.2%)	1 (1.9%)	–	1 (3.8%)	0.635
*Location of the oral pain compliant*					
Upper jaw	21 (23.6%)	13 (24.1%)	2 (22.2%)	6 (23.1%)	0.990
Lower jaw	39 (43.8%)	23 (42.6%)	6 (66.7%)	10 (38.5%)	0.326
Both jaws	10 (11.2%)	7 (13.0%)	–	3 (11.5%)	0.779
Soft tissues	8 (9.0%)	5 (9.3%)	–	3 (11.5%)	0.750
Not an oral complaint: jaw joint or skin	2 (2.2%)	1 (1.9%)		1 (3.8%)	0.635
*Dental visits*					
Registered with local dentist	76 (85.4%)	47 (87.0%)	7 (77.8%)	22 (84.6%)	0.668
Recent dental visit (< 1 year)	67 (75.3%)	42 (77.8%)	5 (55.6%)	20 (76.9%)	0.350
*Transport to local dentist*					
Able to visit local dentist independently	63 (70.8%)	47 (87.0%)^a,b^	4 (44.4%)	12 (46.2%)	< 0.001
Uses local services	11 (12.4%)	4 (7.4%)^b^	–	7 (26.9%)	0.045
Requires assistance from family or caregiver	15 (16.9%)	3 (5.6%)^a,b^	5 (55.6%)	7 (26.9%)	< 0.001
*Elderly experiencing oral pain during the first interview*					
Advised local dentist	19 (21.4%)	10 (18.5%)	1 (11.1%)	8 (30.8%)	0.334
Requires specialist care	2 (2.2%)	1 (1.9%)	1 (11.1%)	–	0.276
Receives specialist care	3 (3.4%)	3 (5.6%)	–	–	0.672
Recent or upcoming dental appointment or no appointment needed	5 (5.6%)	2 (3.7%)	–	3 (11.5%)	0.367

^1^*VAS* Visual analogue scale

^a^ Statistically significant difference (p < 0.05) between robust and frail elderly

^b^ Statistically significant difference (p < 0.05) between robust and complex elderly

Among the participants 10.1% (*n* = 9) could not remember their reported pain complaint and seemed to have no complaints anymore. The most frequently reported pain problems were toothache (23.6%) and denture-related (fitting) complaints (24.7%) **(**Table 2**)**. Minor dental complaints, i.e. complaints that did not require painkillers or urgent dental treatment (such as sensitive teeth), were reported by 23.6% of the elderly. The type of complaints did not differ between respondents with different risk profiles. Most oral pain complaints were related to the lower jaw (43.8%). Of all participants, 9.0% stated their oral complaint was located throughout the oral cavity, no specific location could be determined.

Most elderly participants reported that they were registered at a local dentist (85.4%) and had been visiting their local dentist within the last year (75.3%) for regular dental care. They were often able to visit the dentist independently (70.8%). Robust elderly were in most cases able to go to the dentist independently (87.0%), in contrast to frail (44.4%) and complex elderly (46.2%), who required more assistance from caregivers (*p* < 0.001).

After the interview 21.4% of elderly with oral pain (*n* = 19) were advised to visit a local dentist and 2.2% were advised to return to their dentist and inform if they could be referred to specialist care (*n* = 2), as these elderly had been suffering from oral pain that could not be resolved by their local dentist (e.g. burning mouth problems). All elderly were contacted after 6–8 weeks for a follow-up interview.

### Follow-up interview

The 21 elderly who were advised to visit a dentist or specialist were telephoned for a follow-up interview, of which 19 elderly could be contacted (Table 3). Most of these elderly (*n* = 15, 78.9%) had visited or has an upcoming appointment at their dentist or an oral and maxillofacial surgeon. Only 4 elderly had not visited their dentist. This was because they did not feel the need to visit the dentist and there were other urgent matters.

**Table 3:** Second semi-structured interview

*Second interview (n,%)*	*Total* *(n = 19)*	*Robust* *(n = 10)*	*Frail* *(n = 1)*	*Complex* *(n = 8)*	*P-value between groups*
Still experiencing pain	15 (78.9%)	10 (100%)	1 (100%)	4 (50%)	0.033
VAS^a^ pain (mean, SD)	4.9 (1.8)	4.8 (2.0)	2	5 (1.6)	0.391
Visited a dentist or has an appointment	15 (78.9%)	7 (70%)	1 (100%)	7 (87.5%)	0.675
*Transport to local dentist*					
Able to visit local dentist independently	7 (36.8%)	2 (20.0%)	–	5 (62.5%)	0.303
Uses local services for transport	1 (5.3%)	–	1 (100%)	–	0.091
Required assistance from family or caregiver	2 (10.5%)	2 (20%)	–	1 (12.5%)	0.636
*Reasons for not visiting the dentist*					
No urgency, other (health) problems require more attention	4 (21.0%)	3 (30%)	–	1 (12.5%)	1.000

^a^ VAS: visual analogue scale

## Discussion

The world population is aging and the number of individuals living in community-dwelling elderly has grown [1, 2]. In addition, as a result of improved dental care, edentulism is decreasing and more elderly people are retaining their own teeth [7]. However, frail and care-dependent elderly people are at high risk for oral health problems and pain [10]. Thus far it remains unclear how many community-dwelling experience oral health problems whether they are able to manage their dental care, especially when suffering from oral pain. This study showed that among community-dwelling elderly most reported oral health problems were dry mouth (11.7%) and oral pain (6.2%). Elderly with complex care needs report most frequently oral pain, dry mouth, chewing problems, swallowing problems and a feeling of insecurity. Frail and complex elderly often need assistance of caregivers to visit the dental office.

The prevalence of 6.2% elderly with oral pain differed substantially from the results of Hoeksema et al. [19], who reported a 22% prevalence of oral pain among a comparable group of elderly. The reason for this difference might be related to the difference in the evaluation period during which oral pain was experienced: in the study of Hoeksema et al., this period was ‘during the last 2 years’, [19] while in our study we asked about pain ‘during the last 3 months’. Other studies have shown a prevalence of oral pain and/or oral discomfort at time of the questionnaire ranging from 5.4 to 33.6% among community-dwelling elderly [17, 27].

Another problem with the interpretation of the reported pain prevalence is that most studies do not report symptoms of the oral pain complaint. Only Gluzman et al. [17] provided insight into the symptoms of the complaint. Among their 125 medically-compromised and homebound elderly they found that 15.1% had toothache. This is comparable to the prevalence of toothache in our study (21.3%) which was the second most frequently reported pain complaint, after denture-related pain complaints (24.7%).

Previous studies using these the same risk profiles (robust, frail and complex care needs) among community-dwelling elderly as has been used in our study show similar outcome: frail elderly and elderly with complex care needs show worse general (activities of daily living, quality of life) and oral health outcomes [19, 28] when compared to robust elderly. Other studies have shown similar results among older adults with increasing frailty [27, 29].

Wan et al. studied 200 community-dwelling elderly with orofacial pain [30]. They reported that 10.5% of the community-dwelling elderly could not remember the onset of the pain, comparable to 10.1% of the elderly reporting oral pain in our study who could not remember their oral complaint. This consistent result might be due to an age-related mild memory loss. This means that self-assessment questionnaires given to the elderly should be interpreted with caution because the answers could be biased. Questions on more recent events (e.g., a few weeks in the past) might reduce the risk of such a bias.

This study has shown that 75% of the elderly who live independently at home and feel the need to receive dental care are able to visit the dentist in the Netherlands. Almost 70% of them were still able to visit the dentist independently. The study of Skaar and O’Conner in the US showed that only 46% of community-dwelling elderly yearly visited the dentist [13]. These differences in dental care use may be explained by the fact that in the past when these elderly were young, dental care was provided by the healthcare system in the Netherlands and these elderly are used to regularly visit the dentist. Next, the elderly in our study were suffering from oral pain, which resulted understandably in a higher need for dental treatment and therefore resulted in higher dental care use.

During the second interview, some elderly indicated that they had not visited the dentist or maxillofacial surgeon. The reason for not visiting the dentist or specialist was that elderly felt no need at the moment and had other problems (usually health problems) that required more attention. Similar conclusions were reached in the study of Gaszynska et al. [31] involving care home residents. Elderly who did not visit the dentist within the last 12 months reported that they experienced problems with accessibility, had other major health problems or felt no need to visit the dentist. Their study population lived in a residential care home, which may have affected the high number of elderly reporting difficulties visiting the dentist. Because these elderly did not live independently at home, they required more help with transportation to visit the dental office. This is in contrast to our study population of elderly living independently at home. When our study population grows older and cannot longer live independently at home, they might encounter the same issues with transport to the dentist.

### Strengths and limitations

The strength of this study is the large study population and the focus in this study on oral pain which is thus far hardly described in literature, making this study unique. The limitation is the rather low response rate (45.9%). These relatively low response rates are commonly seen in elderly research projects [32–34]. It is most likely that the elderly who did not return the questionnaire and did not participate in this study are older, more frail and have higher needs for complex care. It is very likely that these community-dwelling elderly also have more oral health problems, more oral pain complaints and more problems visiting the dentist.

Elderly are facing often many difficulties with their health. It seems that oral health in the elderly population when compared to other big health issues is not a first a priority [35]. Another possible limitation is the use of self-assessment questionnaires and structured interviews to assess oral health. Even though the structured interview was performed by a researcher who was a dentist, no intraoral examination was conducted, which means that the reported symptoms could not be confirmed clinically.

## Conclusion

Dry mouth and oral pain are most reported oral health problems among community-dwelling elderly. Elderly with complex care needs report most oral health problems. In case an elderly seeks dental treatment to alleviate an oral pain complaint, most elderly in this study were able to organize dental care and transport to the dentist. Frail and complex elderly often need assistance from caregivers to visit the dentist.

### Clinical significance

As long as elderly live independently at home and feel a personal need to receive dental care, they are able to manage their dental care. Elderly with complex care needs report more oral health problems. Therefore care-takers should keep in mind that when frailty progresses, visiting the dentist may become more and more difficult and the risk for poor oral health increases.

## Data Availability

The datasets generated and/or analyzed during the current study are not publicly available due to the fact that the data contains personal information and comments, which are necessary for correct interpretation of oral pain complaint. Datasets are available from the corresponding author on reasonable request.

## Abbreviations

GFI: Groningen Frailty Indicator
IM-E-SA: Intermed for the Elderly Self-Assessment
GP: General practitioner
